# Paradoxical worsening of bradycardia following atropine administration

**DOI:** 10.29045/14784726.2022.09.7.2.38

**Published:** 2022-09-01

**Authors:** Richard Armour, Charmane Learning, Jan Trojanowski

**Affiliations:** Ambulance Victoria; Monash University; Charles Sturt University; British Columbia Emergency Health Services; Columbia Paramedic Academy; British Columbia Emergency Health Services; Vancouver Coastal Health; University of British Columbia

**Keywords:** adverse event, atropine, bradycardia, paramedic

## Abstract

**Introduction:**

Bradyarrhythmias are a common entity in both emergency and out-of-hospital (OOH) medicine. In unstable bradycardic patients, paramedics will often initiate life-saving therapies in the OOH setting. Clinical guidelines for bradyarrhythmias are largely consistent across the globe, with intravenous (IV) atropine recommended as a first-line therapy, escalating to IV adrenaline or isoprenaline and transcutaneous pacing where atropine is unsuccessful. In this case report, we describe a case in the OOH setting of ventricular standstill following the administration of atropine to a patient with bradycardia and 2:1 heart block.

**Case presentation:**

The patient was a 77-year-old female presenting with a symptomatic 2:1 heart block. Following a single dose of 600 micrograms IV atropine, the patient deteriorated into ventricular standstill with a loss of consciousness and decorticate posturing. The patient was successfully managed with an IV infusion of adrenaline and subsequently received an implanted pacemaker in hospital.

**Conclusion:**

The paradoxical worsening of this patient’s bradycardia following atropine administration may have been related to the location of the heart block. It has been shown that patients with atrioventricular blocks at the level of the His-Purkinje fibres (infranodal) are at an increased risk of adverse events following atropine administration, while those at the nodal level or secondary to increased vagal tone are more likely to respond favourably. Paramedics should be prepared to manage unexpected adverse events secondary to atropine administration in patients with heart block.

## Introduction

Bradyarrhythmias are a common entity in both emergency and out-of-hospital (OOH) medicine (Abrich et al., 2020; Deal, 2013; Schwartz et al., 2004). These presentations may range from the relatively benign to the dangerously unstable requiring emergency interventions to prevent death. In unstable bradycardic patients, paramedics will often initiate life-saving therapies in the OOH setting (Beygui et al., 2020). Clinical guidelines for bradyarrhythmias are largely consistent across the globe, with intravenous (IV) atropine recommended as a first-line therapy, escalating to IV adrenaline or isoprenaline and transcutaneous pacing where atropine is unsuccessful (Kusumoto et al., 2019; Soar et al., 2015). Although paradoxical worsening of bradyarrhythmias has previously been described following atropine administration in cardiac transplant recipients (Bernheim et al., 2004; Ji et al., 2013), only sporadic case reports detail worsening of bradyarrhythmias in patients without previous significant cardiac disease in the OOH setting. In this case report, we describe a case in the OOH setting of ventricular standstill following the administration of atropine to a patient with bradycardia and 2:1 heart block.

## Case presentation

The patient was a 77-year-old female with a past medical history significant for hypertension, asthma and a previous hip replacement. At the time of the event, the patient’s only medication was Ramipril 5 mg per day and she lived independently in her own home. The patient had not recently altered any of her medications, changed her diet or lifestyle or used drugs or alcohol preceding the event.

At approximately 14:30 on the day of the event while out walking, the patient experienced a sudden onset of dizziness, nausea and vomiting and experienced multiple pre-syncopal episodes at rest and on exertion. The patient was able to return to her home but after laying flat experienced a syncopal episode each time she tried to sit up and so 9-1-1 was called at 15:59. A dual-crewed primary care paramedic unit and a dual-crewed advanced care paramedic unit were dispatched to the scene given the symptoms described during the 9-1-1 call (Supplementary 1).

**Figure 1:**
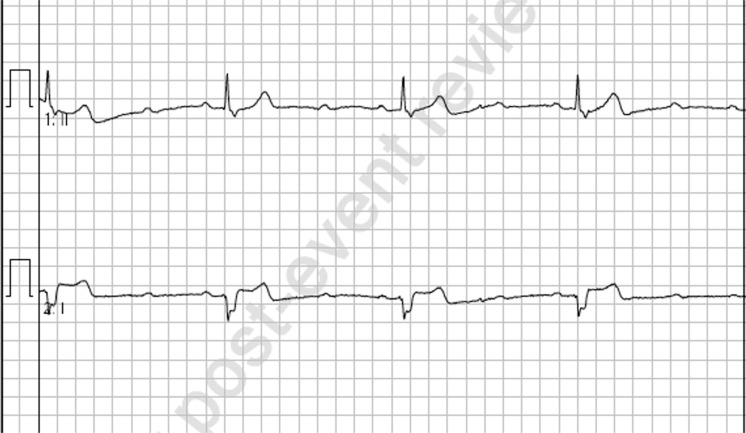
Figure 1. Initial rhythm tracing.

**Figure 2:**
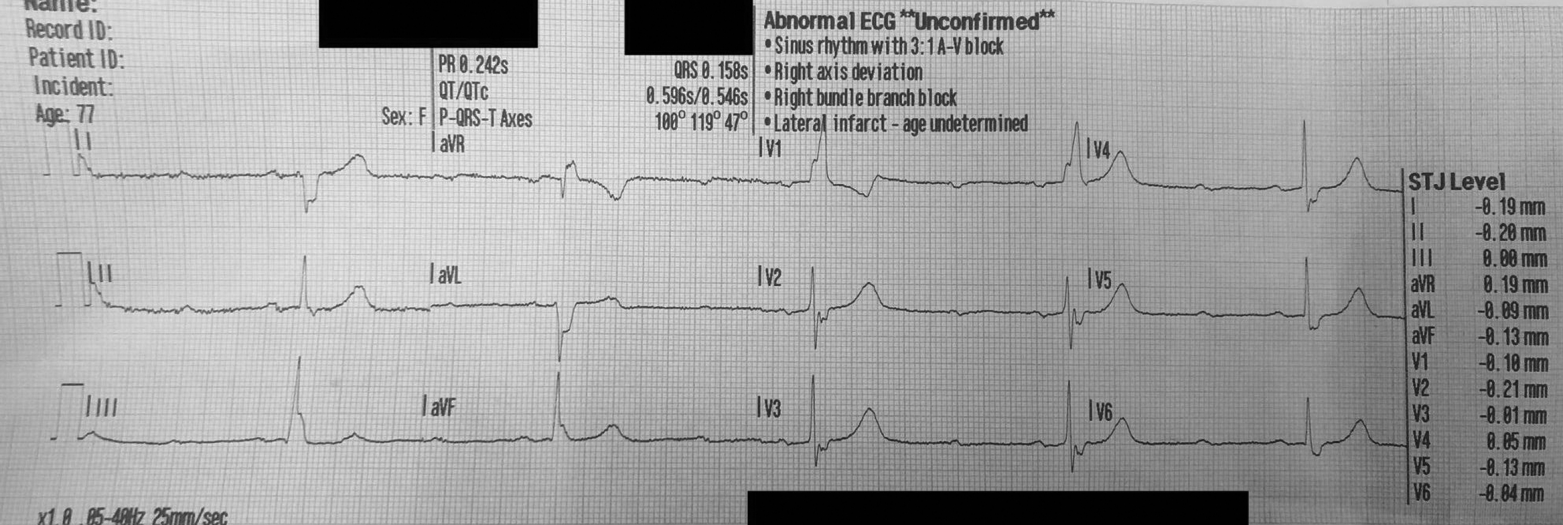
Figure 2. Initial 12-lead electrocardiogram.

On arrival to the scene, the patient was found supine in bed, alert and oriented with weak peripheral pulses at a rate of 25 and cool, clammy skin. On any attempt at movement, the patient would again become pre-syncopal and so was left in her current position during assessment. The initial vital signs are found in Table 1 and the initial rhythm strip and 12-lead electrocardiogram (ECG) revealed a 2:1 heart block (Figures 1 and 2).

**Table 1.** Patient vital signs.

Vital signs	Pre-atropine	Post-ventricular standstill	Post-adrenaline infusion	At hospital
**GCS score**	15/15 (4,5,6)	14/15 (4,4,6)	15/15 (4,5,6)	15/15 (4,5,6)
**Respiratory rate (breaths/min)**	16	24	20	20
**SpO2**	99% (RA)	99% (RA)	99% (RA)	99% (RA)
**Heart rate (beats/min)**	25	25	47	45
**Blood pressure (mmHg)**	140/28	88/44	110/42	115/39

GCS: Glasgow Coma Scale.

**Figure 3:**
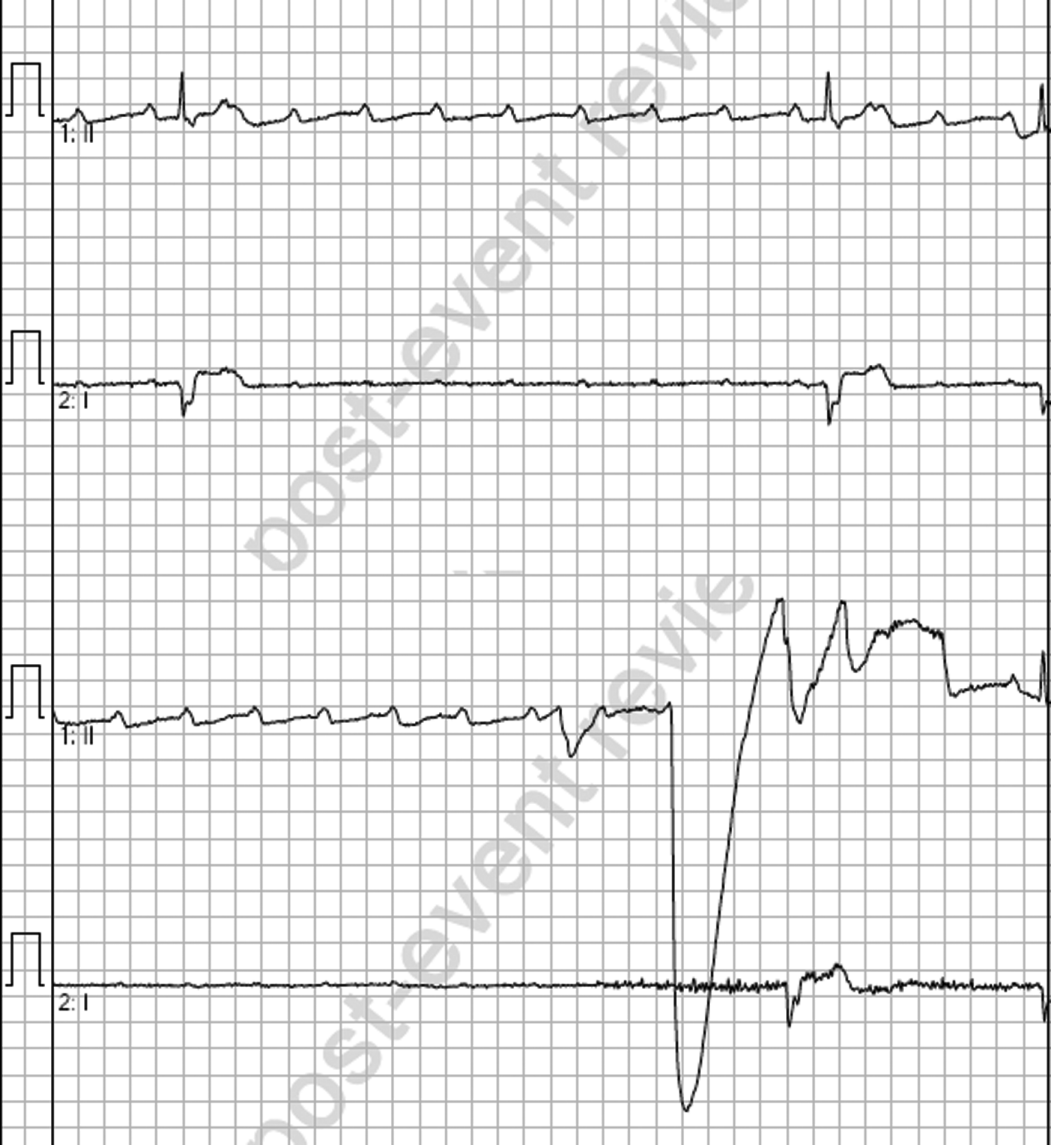
Figure 3. Slowing of ventricular conduction post-atropine.

**Figure 4:**
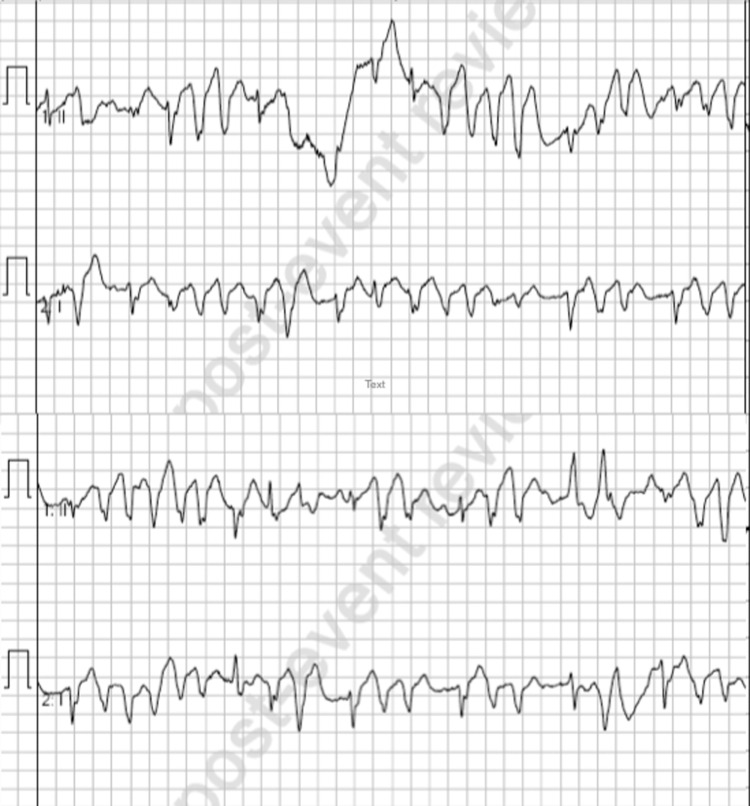
Figure 4. Ventricular tachycardia post-adrenaline.

A single dose of 600 micrograms IV atropine was administered and within one minute of this administration the patient experienced a period of ventricular standstill (Figure 3) lasting approximately 30 seconds until therapy was commenced, accompanied by loss of consciousness, decorticate posturing and urinary incontinence. This episode was treated with a single dose of 50 micrograms IV adrenaline (prepared as 10 micrograms/mL), which led to a short episode of pulsatile, self-terminating polymorphic ventricular tachycardia and a subsequent return to the bradycardic 2:1 heart block (Figure 4). With the patient’s symptoms worsening and precluding extrication, an adrenaline infusion was commenced at 2 micrograms/min and subsequently escalated in 2 micrograms/min increments until a response was achieved at 10 micrograms/min approximately 10 minutes after initiation of the infusion (Table 1). A follow-up 12-lead ECG is not available as the patient had at this time been transitioned to defibrillator pads for monitoring; however, the rhythm remained a 2:1 heart block at an elevated rate for the remainder of patient care.

The patient was conveyed to hospital with the on-going adrenaline infusion, having experienced a reduction in the dizziness and nausea and with no further pre-syncopal or syncopal episodes. Given these improvements and the patient experiencing no side effects from the adrenaline infusion, a transition to transcutaneous pacing was not considered to be required. The patient subsequently received an implanted pacemaker and was later discharged back to the community.

## Discussion

This case report discusses the management of a patient with symptomatic 2:1 heart block treated in the OOH setting with IV atropine and subsequently adrenaline when the patient experienced a paradoxical worsening of bradyarrhythmia secondary to atropine administration.

Current clinical guidelines produced by the European Resuscitation Council (ERC) (Perkins et al., 2021) and American Heart Association (AHA) (Kusumoto et al., 2019) recommend the use of IV atropine as a first-line agent in the management of symptomatic bradyarrhythmias, including heart block. ERC guidelines acknowledge the risk of high-grade atrioventricular (AV) block following cardiac transplantation, while the AHA guidelines explicitly state that atropine is likely only useful in AV block at the AV nodal level or secondary to increased vagal tone (Kusumoto et al., 2019). The AHA guidelines specifically reference an increased risk of adverse events with the use of atropine at the level of the His-Purkinje system (infranodal) (Kusumoto et al., 2019).

The differentiation of AV block as nodal or infranodal may be difficult in the OOH setting utilising only the ECG, though. Conventional teaching suggests that in patients with 2:1 heart block and wide QRS complexes, such as the complexes in this case, an infranodal block is likely present (Kantharia & Shah, 2013), and thus they are unlikely to respond to atropine and are more likely to experience adverse events secondary to atropine administration. However, up to 20% of patients with 2:1 block and wide QRS complexes may actually be experiencing a block at the nodal level and may therefore benefit from atropine administration (Barold, 2001; Kusumoto et al., 2019). The presence of a nodal block, rather than infranodal block, in a patient with 2:1 heart block may be implied by the presence of a prolonged PR interval and narrow QRS complex which improves following atropine administration (Bhargava & India, 2012). In this case, given the patient presented in a 2:1 heart block with normal PR intervals and wide QRS complexes, it was retrospectively considered likely the patient was suffering with an infranodal block and thus experienced a paradoxical worsening of her bradyarrhythmia secondary to atropine administration.

## Conclusion

The administration of atropine in the OOH setting is a common intervention for bradyarrhythmias and is often thought to have few significant side effects in the context of managing life-threatening bradycardia. In the case presented, however, the administration of atropine in a patient with a symptomatic 2:1 heart block resulted in ventricular standstill requiring the administration of adrenaline. Paramedics should be aware of ECG features suggestive of both nodal and infranodal 2:1 heart blocks and be prepared to manage adverse events following atropine administration accordingly.

## Author contributions

RA and CL attended the case and prepared the manuscript for publication. JT assisted in manuscript preparation and patient follow-up. RA acts as the guarantor for this article.

## Conflict of interest

None declared.

## Ethics

The Provincial Health Services Authority (PHSA) Ethics Service provided input on the conduct of this case study and assisted in determining that Research Ethics Board Approval was not required. The patient has provided informed and signed consent relevant to the publication of this article.

## Funding

None.
